# The new workplace II: protocol for a prospective full-panel registry study of work factors, sickness absence, and exit from working life among Norwegian employees

**DOI:** 10.1186/s40064-016-1896-z

**Published:** 2016-03-01

**Authors:** Morten Birkeland Nielsen, Solveig Christiansen, Anne-Marthe Rustad Indregard, Jan Shahid Emberland, Shahrooz Elka, Stein Knardahl

**Affiliations:** National Institute of Occupational Health, PB 8149 Dep, 0033 Oslo, Norway

**Keywords:** Work exposure, Health and well-being, Mediators and moderators, Sickness absence, Disability

## Abstract

**Background:**

Previous studies on the effects of work factors on absence and disability retirement have only addressed a limited set of factors and little is known about the mechanisms that govern relationships between work exposures and sickness absence/disability retirement. The main aims of the present project are (1) to examine the impact of a comprehensive set of psychological, social, organizational, and mechanical work factors work factors on sickness absence and disability retirement, and (2) to identify moderating and mediating variables that determine how and when exposures at the workplace are related to sickness absence and disability retirement.

**Methods:**

The study design is prospective and based on longitudinal survey data linked to registry data on sickness absence and disability. Altogether 14,501 respondents have given their permission to the linking of their survey questionnaire data to registry data. The project has been approved by the Regional Committees for Medical and Health Research Ethics and has permission from The Norwegian Data Protection Authority. The questionnaire instruments contain psychometrically validated items and inventories on demographic background factors, work exposures, individual dispositions and attitudes, somatic health, mental distress, well-being, lifestyle factors, and work ability.

**Discussion:**

The findings will have relevance for, and benefit working life and the larger society in a number of ways. Firstly, it will lead to a more knowledge about which work factors that contribute to health, sickness absence, and participation in/exit from the labour force. Secondly, a better understanding of which mediators and moderators that modify and govern these relationships. Both are central to the development of laws and regulations and to any political decision on measures to tackle sickness absence and early retirement.

## Background

Sickness absence and disability retirement can be considered as indicators of health status, and as markers of social, psychological and physical functioning (i.e. work ability) for the working population (Niedhammer et al. 2013; Marmot et al. 1995; Kivimaki et al. 2004). Based on increasing life expectancies one should assume that the overall health status of the Norwegian population is better than ever. It is therefore a paradox that the prevalence of health problems, absence, and disability pension seems to be maintained. For instance, findings show that subjective health complaints (like musculoskeletal complaints, fatigue, depression, mental distress) are as prevalent as before (Indregard et al. 2013; Kjeldsberg et al. 2013). As for sickness absence, estimates from Statistics Norway shows an adjusted absence rate of 5.8 % in the second quarter of 2015.^1^ The rates in Norway are among the highest in Northern Europe and levels of absence have been found to be constant during the last 5 years (Krane et al. 2014; MoHaC Services 2014). The economic burden of sickness absence is considerable and the authorities want to reduce these costs (Krane et al. 2014; Strømholm et al. 2015).

Although certified sickness absence in some cases may be issued even if the formal criteria are not satisfied (e.g., due to difficult working conditions such as interpersonal conflict and workplace bullying), the eligibility criteria for sickness absence and disability benefits in Norway are strictly medical. Consequently, to be able to develop and implement sound and effective measures that can reduce sickness absence and disability retirement, one needs to establish and understand factors that promote and inhibit both health and work ability. A recent study of sickness absence in 31 countries in Europe concluded that preventive measures should take psychosocial work environment more comprehensively into account in order to reduce sickness absence and improve health at work (Niedhammer et al. 2013). This suggests that the identification of work factors that influence health and work ability, together with the mediating and moderating variables that can explain how and when work factors have an impact on individuals, are especially beneficial for reducing the rates of health problems, sickness absence and disability retirement.

Several studies confirm that psychological, social, organizational, and mechanical work factors contribute to employees’ health in the form of musculoskeletal complaints (Christensen and Knardahl 2010, 2012; Ariens et al. 2001), headache (Tynes et al. 2013; Christensen and Knardahl 2012), cardiovascular disease (Niedhammer et al. 1998; Kuper and Marmot 2003), and mental distress (Finne et al. 2014; Johannessen et al. 2013; Nielsen et al. 2012). Working conditions has also been related to indicators of work ability such as motivation (Conchie 2013), sickness absence (Niedhammer et al. 2013; Sterud 2014; Lund et al. 2006) and disability and early retirement (Appelberg et al. 1996; Blekesaune and Solem 2005). The relative importance of work factors on absence was demonstrated in a meta-analytic study on antecedents of general absence which showed that work-related factors were better predictors of absence than psychological and demographic correlates (Michie and Williams 2003).

The potential impact of work factors on absence was further substantiated in a systematic review of work factors associated with sickness absence which found that absence was mainly predicted by long hours worked, work overload and pressure, lack of control over work, lack of participation in decision making, poor social support, and unclear management and work role (Michie and Williams 2003). As for disability retirement, systematic reviews of psychosocial factors at work and disability retirement highlights that only a few factors have been studied in previous prospective research. Of the variables which have been examined, the systematic reviews show that low control, monotonous work, job strain, effort-reward imbalance, a lack of social support, problems related to the organization of work and to leadership behaviors are related to an increased risk of disability (Knardahl et al. submitted; Dragano and Schneider 2011).

While it has been established that at least some exposure factors in the work environment are related to sickness absence and disability retirement, the vast majority of studies have only examined direct relationships between work-related variables and exit from working life. It has, however, been claimed that merely presenting factors associated with sickness absence does not provide a sufficient explanation for how the variables are related (Kristensen 1991). With the exception of some studies on the job demands—control model and effort—reward—imbalance model (e.g., Ala-Mursula et al. 2005), there are few studies which have examined interactions between different predictors. Consequently, to this date there is a significant lack of research on the mechanisms that can explain how, and under which conditions, work exposures can have an impact on participation in, or exit from, working life (Allebeck and Mastekaasa 2004).

Steers and Rhodes (1978) claimed that attendance is directly influenced by two primary factors: (a) attendance motivation, and (b) ability to come to work. Attendance motivation, in turn, is largely influenced by (a) satisfaction with the job situation, and (b) various internal and external pressures to attend. This suggests that absence is determined by many different individual and situational variables and that knowledge about sickness absence and disability retirement is dependent upon a wide variety of factors, as well as their interactions. For instance, illness perceptions combined with a general physical inactivity may contribute to a reduction of work ability in many individuals. A model stating that “fear avoidance” and catastrophizing (the belief that pain will not go away and that any attempts will make pain worse) are significant mechanisms of disability, has gained wide acceptance (Leeuw et al. 2007). Expectancies, cognitive appraisal of complaints/symptoms, pain beliefs) is receiving increasing attention in research on chronification of complaints and disability (Amanzio et al. 2009).

Theoretically, sickness absence can also be explained in the light of the conservation of resources theory (COR; Hobfoll 1989). Study findings indicate that personal resources (e.g., perceived control, self-efficacy, perceptions of improvement) and social resources (e.g., emotional support, assistance from friends and family) buffer against the potential negative impact of stressful life events (Folkman and Moskowitz 2004; Lazarus and Folkman 1984). COR theory extends prior theories, such as the transactional theory of stress (Lazarus and Folkman 1984), by acknowledging that strain stems from the combined effect of the subjective perception of an event as taxing or exceeding available resources and the objective or actual environmental circumstances that threaten or cause depletion of people’s resources (Alvaro et al. 2010). The fundamental proposition of the COR is that individuals’ well-being, work attitudes, and work behaviors are dependent on their perceived access to resources. When valued resources are threatened or lost as a result of exposures at the workplace, employees may actively strive to prevent further resource loss by withdrawing from work, and absence from work can thereby be understood as a response to perceived or actual loss of resources under conditions of chronic work stress (Nguyen et al. 2013).

Taken together, previous research have established psychological, social, organizational, and mechanical work exposure as potential predictors of sickness absence and disability retirement. As the evidence for the examined work factors varies across studies, there is, however, a need for further research on the relative impact of different work exposures, as well as for establishing novel work factors that may influence the outcome variables. In addition, little is known about the mechanisms and conditional factors that explain how and when work exposures are related to sickness absence and disability retirement. The Norwegian official report NOU 2010:13: “Work for health. Sickness absence and exit from work in the health—and care sector” concluded that “in spite of research efforts in this field, there is inadequate knowledge of causes of sickness absence and exit from working life to health-related compensation. Most of the available research is based on observation studies in which methodological weaknesses are ground for caution in the interpretation of the results” (p. 120).^2^

**Fig. 1:**
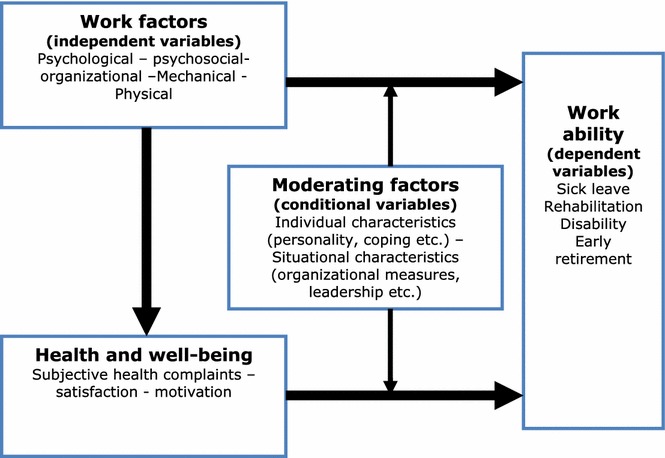
Expected relationships between study variables

In contrast to previous studies, the present project measures a battery of specific factors at individual-, group, and organizational-level and tracks the individuals over time to attain a more reliable characterization of work factors, attitudes, subjective health, and changes and reorganization. Data on sickness absence and disability retirement are obtained from official registries. As depicted in Fig. 1, we expect that the impact of work exposures on sickness absence and disability retirement is mediated through health complaints, well-being, and job satisfaction and that both the direct and indirect association between work exposures and the outcome variables are moderated by individual and situational characteristics. The present paper describes the research questions, study design and data collection methods in this planned study.

## Research questions

The following primary research questions will be addressed in the project through the use of prospective studies and registry data on sickness absence and disability retirement:

1. Which work factors (psychological, social, organizational, and mechanical) are risk factors for subsequent sickness absence and disability retirement?
2. Which health complaints are risk factors for subsequent sickness absence and disability retirement?
3. What are the individual and occupational factors that moderate the associations between work stressors, health outcomes, and sick leave and disability retirement?
4. Do subjective health complaints, distress, general well-being, job satisfaction, and motivation mediate the association between exposure to work factors and sickness absence and disability retirement?

## Methods

### Ethical approval

This project has been approved by the Regional Committees for Medical and Health Research Ethics (REC) in Norway (REC South East), has permission from The Norwegian Data Protection Authority, and was conducted in accordance with the World Medical Association Declaration of Helsinki. All study participants provided their informed consent. Data were collected through the *Resource Center for psychological and social factors at work*, developed by The National Institute of Occupational Health (NIOH). This is a web-based system for secure administration of questionnaires. The system is developed for the purpose of tracking individuals over time and to couple data to registries in a way that satisfies demands for anonymity and personal security. When accessing the web-based questionnaire by a personal login code, informed consent had to be confirmed before responding to the questionnaire. This consent procedure was considered as equal to a written informed consent. The procedure was approved by The Norwegian Data Protection Authority and REC. Respondents are treated anonymously in the data analyses. Only respondents who actively (by response) permitted the linking of their answers to registries are included in the present project.

### Study design

The current project is an extension of the longitudinal survey study “The new work place: work, health, and participation in the new work life” at NIOH Norway (see Christensen and Knardahl 2010; Finne et al. 2014; Emberland and Knardahl 2015). The general aim of this comprehensive project is to obtain new knowledge about working conditions of consequence to employee health and work ability over time. The project described in this study protocol extends the mother project by linking already collected survey data to national registry data on sickness absence and disability retirement.

The survey part of this project is based on data from a large sample of Norwegian adults employed in a full time or part time position. Subjects were recruited from organizations in Norway that were contacted and offered to participate in the study. In return for participating and making the data available for research, companies received written reports and oral presentations of results as a tool for organizational development and an aid for monitoring the organizational work environment. The survey was web-based, although participants with limited access to computers at work were given the option of filling out a paper version of the questionnaire. Employees and management in the companies were informed at the organizational level first and the participating enterprises were required to return an organization level questionnaire which asked about demographic information about the enterprise. Subsequently, all employees excluding those on sick leave were mailed a letter with information about the survey. This letter contained either a personalized code for logging into the web-questionnaire or a paper version of the questionnaire with a pre-stamped return envelope, in addition to information about the survey. The written information explained the aims of the study and assured that responses would be treated confidentially, in strict accordance with the general guidelines and specific license from The Norwegian Data Protection Authority. Employees were given the opportunity of filling out the questionnaire at work, but it was also possible to fill it out from home or any other location. Each subject had the opportunity to log into the web-questionnaire an unlimited number of times to change or complete their answers during the survey period.

From November 2004 to December 15th 2014, a total of 31,823 employees recruited from 97 organizations have been invited (at least once) to participate in the survey. Altogether 15,282 persons responded (response rate: 48 %). Of these, 14,501 respondents permitted linking survey questionnaire data to official registry data (acceptance rate: 95 %). By the same date, 65 organizations and 14,586 persons had been invited to participate in the second survey assessment. A total of 7199 (49 %) participated in the second assessment. The average time-lag between the survey measurement points was 24 months (range 17–36 months).The survey data were linked to registry data on sickness absence and disability retirement obtained from the Norwegian Labour and Welfare Service.

### Respondents

The study sample is based responders who accepted that their responses would be linked with national registries on social security benefits. The organizations, which employees were recruited from, represented a wide range of occupational sectors including healthcare, education, government and public administration, engineering, business and industry. Participating organizations provided a list of employees’ departmental affiliation, home address and occupational title according to the Norwegian Standard Classification of Occupations (STYRK), developed by Statistics Norway and based on the International Classification of Occupation (ISCO-88). About 85 % of the sample responded to the survey using the electronic survey form.

Demographic characteristics for the sample are presented in Table 1. Mean age in the total baseline sample was 42.75 (SD = 10.8) years with a range from 16 to 72. The sample consisted of more women (55.7 %) than men (44.3 %). About 4 % had between 1 and 9 years of education, 33 % had between 10 and 12 years, 44 % had between 13 and 16 years and 20 % had 16 years or more. The majority of the sample reported to be in regular full time employment (91 %). About 45 % of the respondents were on daily working time arrangement, 32 % had a fixed schedule, and 19.6 % worked in shift-arrangements. The three largest occupation groups among all employees in the sample were professionals (28.8 %), technicians and associate professionals (27.5 %), and service workers and shop and market sales workers (23.5 %). Altogether 20.4 % had a leadership position. The overall sample characteristics suggest that the sample is quite heterogeneous.

**Table 1:** Demographic characteristics of the study sample

	Respondent who provided consent to use registry data (n = 14,501)	
	N	%
Age		
<30	1828	12.6
30–39	4072	28.1
40–49	4375	30.2
50–59	3300	22.8
≥60	926	6.4
Gender		
Male	6425	44.3
Female	8076	55.7
Marital status		
Not married	1818	16.2
Married	6119	54.6
Cohabiting	2332	20.8
Widowed	89	0.8
Divorced	664	5.9
Separated	180	1.6
Educational level		
<9 years	390	3.8
10–12 years	3304	32.5
13–16 years	4423	43.5
16 years<	2043	20.1
Employment		
Regular full time employment	10,693	91
Time-limited contract	631	5.4
On call staff/	352	3.0
Other	70	0.6
Classification of occupation		
Legislators, senior officials and managers	1222	8.8
Professionals	3971	28.8
Technicians and associate professionals	3799	27.5
Clerks	762	5.5
Service workers and shop and market sales workers	3245	23.5
Craft and related trades workers	425	3.1
Plant and machine operators and assemblers	50	0.4
Elementary occupations	135	1.0
Armed forces and unspecified	199	1.4
Work schedule		
Fixed day-time arrangement	6232	44.9
Flexible working-time arrangement	4451	32.1
Fixed evening-arrangement	291	2.1
Fixed night-time arrangement	151	1.1
Shift work arrangement	2719	19.6
Leadership responsibility		
No	8381	79.6
Yes, middle-level manager	1892	18
Yes, top-level manager	258	2.4

### Questionnaire instruments and items

All participating organization were required to return a questionnaire which contained information about type of organization (public vs private, national vs multinational), line of business, number of units/departments, number of employees in organization and within each unit/department, work tasks, financial situation, recent organizational changes, turnover rates, available human resource measures, and whether the organization have a cooperation agreement regarding a more inclusive working life (IW-agreement).^3^

The questionnaire which were distributed to the individual employees contained items and inventories which can be classified into the following five main categories:

1. *Background information* Demographic factors, education and skills, characteristic of the job, travel distance to work, use of home office, overtime work.
2. *Psychological, social, organizational, and mechanical work factors* Job demands, role conflict and ambiguity, decision latitude, predictability in work tasks, mastery of work, social support, workplace bullying and harassment, fair and empowering leadership, organizational climate, work-family conflicts, job centrality, commitment, group work, work motivation, job satisfaction, computer work, physical labor, social interactions with clients including emotion work (i.e. emotional dissonance and positive display), organizational changes and restructuring.
3. *Individual characteristics and attitudes* Self-leadership, self-efficacy, dispositional optimism, the five factor model of personality (extraversion, agreeableness, conscientiousness, neuroticism, openness), coping strategies, attitudes towards work including intent to leave and sickness presenteeism.
4. *Subjective health complaints and mental distress* Headache, neck pain, pain in shoulder and overarm, pain in lower arm, wrist and hands, back pain, chest pain, pain in feet, nausea and vomiting, stomach pain, gastritis, palpitations, eczema and rash, eyestrain, difficulty breathing and asthma, coughing, cold, tiredness, dizziness, worry, depression, anxiety, general tension, sleep problems, mental distress, fatigue, psychotropic drug use.
5. *Life style factors and work ability* Smoking, alcohol use, physical activity, work ability.

### Psychological, social and mechanical work factors

Psychological, social, and mechanical work factors were assessed by the General Nordic Questionnaire for Psychological and Social Factors at Work (QPS_Nordic; Dallner et al. 2000). QPS_Nordic_ has been thoroughly tested for validity and reliability and has shown good psychometric properties (Dallner et al. 2000; Wannstrom et al. 2009). The following fifteen scales were studied; quantitative demands (i.e. time pressure and amount of work), decision demands (i.e. demands for decision-making and attention), decision control (i.e. influence on decisions regarding work tasks, choice of coworkers, and contacts with clients), control over work intensity (i.e. influence on time, pace, and breaks), role conflict (i.e. conflicts between demands and resources, conflicting requests), role clarity (i.e. clarity of goals and objectives at work), support from immediate superior (i.e. instrumental and emotional support, and appreciation), empowering leadership (i.e. encouragement for participation in important decisions and expressing differing opinions, development of skills), fair leadership (i.e. distribute work fairly and treat workers fairly and equally), predictability during the next month (i.e. predictability of tasks, coworkers, and superiors), predictability during the next 2 years (i.e. predictability of job security and learning demands), commitment to organization (i.e. positive feelings and attitudes towards the workplace), social climate (i.e. whether the social climate is encouraging/supportive, distrustful/suspicious, relaxed/comfortable), positive challenge at work (i.e. usefulness of skills and knowledge, meaningfulness of work, and if work is challenging in a positive way), and human resource primacy (i.e. organizational practices pertaining to rewarding workers for well-done jobs, taking good care of workers, the interest of management in the health and well-being of workers). The scales varied from three to five items. Response scale for the QPS_Nordic_ items was: “1 = very seldom or never”, “2 = somewhat seldom”, “3 = sometimes”, “4 = somewhat often”, and “5 = very often or always”. Exceptions were commitment to organization with the response alternatives: “1 = disagree totally”, “2 = disagree to some extent”, “3 = indifferent”, “4 = agree to some extent”, and “5 = agree totally” and predictability during the next 2 years, human resource primacy, and social climate: “1 = very little or not at all”, “2 = rather little”, “3 = somewhat”, “4 = rather much”, and “5 = very much”.

Three single items from QPS_Nordic_ were also included. “Are there rumors concerning changes at your workplace?” with the response scale “1 = very seldom or never” to “5 = very often or always”. Workplace bullying was measured by presenting the respondent with a formal definition of the bullying construct and then asking: “Have you noticed anyone being subjected to harassment or bullying at your workplace during the last 6 months?” and “Have you been subjected to bullying or harassment at the workplace during the last 6 months?” The response categories for both items were “yes” and “no”. A single question measured organizational procedural justice related to organizational change: “Procedures are designed to hear the concerns of all those affected by the decision” with the response alternatives “1 = strongly agree”, “2 = quite agree”, “3 = neutral”, “4 = quite disagree”, and “5 = strongly disagree”.

Emotion work was measured with four items tapping emotional dissonance (example item: “How often does it occur in your job that one has to display positive emotions that do not correspond to what is felt in this situation?”, and four items tapping positive emotions (example item: “In your job how often does it occur that you have to display pleasant emotions towards your clients?”). These items were taken directly from the Frankfurt Emotion Work Scale (Zapf et al. 1999). Responses were provided on a five point scale with the following response alternatives ranging from “Seldom or never”, “Once per week”, “Once per day”, “Several times per day”, and “Several times an hour”.

Sixteen questions about changes at the workplace during the last 12 months, with “Yes” and “No” as response alternatives were used to assess work-related changes. This part of the questionnaire did also include questions about the perceived impact of organizational and work-related changes on the employees and resources spent on adapting to the changes. Rumors of change were assessed with five items asking the respondents how often during the last 12 months they had discussed the possibility of downsizing, reorganization, outsourcing, or shutdown with their colleagues. Responses were provided on the following scale “1 = very seldom or never”, “2 = somewhat seldom”, “3 = sometimes”, “4 = somewhat often”, and “5 = very often or daily”.

### Individual characteristics and attitudes

Several measures of individual characteristics and attitudes were included in the questionnaire. Dispositional optimism, the generalized expectation of positive rather than negative outcomes in life, was measured with three items from the “Revised Life Orientation Test” (LOT-R; Scheier et al. 1994), an instrument developed to measure individual differences in optimism versus pessimism. Self-efficacy was assessed with three items from the Generalized Self-Efficacy scale (GSE; Schwarzer and Jerusalem 1995). Response categories for the LOT-R and GSE scales were 1 = “Strongly disagree”, 2 = “Disagree”, 3 = “Neutral”, 4 = “Agree”, and 5 = “Strongly agree”. Personality was measured with an abbreviated version of the International Personality Item Pool (Nielsen and Knardahl 2015a; Goldberg 1999). This Big-Five personality marker consists of 15 items measuring extraversion, agreeableness, conscientiousness, neuroticism, and openness (Nielsen and Knardahl 2015a). The participants rated each item on a seven-point Likert scale (from “Very Inaccurate” to “Very Accurate”). Coping strategies were examined with a Norwegian translation of the 28 item Brief COPE inventory (Carver 1997; Nielsen and Knardahl 2014) which is an abridged version of the full COPE inventory (Carver et al. 1989). Respondents indicate the extent to which they agree or disagree with the items, and responses to all items are made by means of a four point scale ranging from “Usually I do not do this at all” to “Usually I do this a lot”. Sickness presenteeism was measured with single item question asking “During the last 12 months, how may working days have you gone to work even though you were ill?” (Johns 2010).

### Subjective health complaints, mental distress, and well-being

Somatic health complaints were measured by 21 single item questions asking “have you been bothered by … “neck pain”, “headache”, “back pain” etc. during the last 4 weeks” (Steingrimsdottir et al. 2004), with optional answers “not bothered” (1), “a little bothered” (2), “rather intensely bothered” (3), and “very intensely bothered” (4). Mental health was measured by the ten items version of the Hopkins Symptom Checklist (HSCL-10) (Derogatis et al. 1974). The HSCL-10 consists of ten items on a four-point scale, ranging from “1 = not at all” to “4 = extremely”. Physical and mental fatigue was measured by six items on “personal burnout” from the Copenhagen Burnout Inventory (Kristensen et al. 2005). The five point response scale ranged from “Never/almost never” through “Once or twice a month”, “Once or twice a week”, and “Three to four times a week” to “(Almost) every day”. The two aspects of troubled sleep that were measured were (1) difficulties initiating sleep and (2) disturbed sleep (Vleeshouwers et al. 2015). The question was worded as follows: “Have you experienced the following symptoms in the last 4 weeks?” with the following two items: ‘difficulties falling asleep’ and ‘disturbed sleep’. Response alternatives were: “0 times”, “1–3 times per month”, “1–2 times per week”, “3–5 times per week”, and “6–7 times per week”. Work ability was assessed with the work ability index (WAI) which comprises seven items (Ilmarinen et al. 1997). The WAI intends to capture both subjective evaluations to work ability, work impairment, and objectively verifiable information on health status.

### Registry data

Having secured informed consent from participants, survey data were linked to the sickness and disability benefit register of the Norwegian Labour and Welfare Administration by the use of the unique 11-digit national identity number. The national identity number is used in all kinds of contact with the authorities such as paying tax, registering at a new address and applying for social security benefits. The registers provide complete records of disability retirement and all sickness absence episodes—including the length and medical diagnosis—which are compensated by the national insurance sickness benefit (Strømholm et al. 2015). As a general rule, everybody working or living in Norway is covered by the Norwegian national social insurance. Sickness benefit under the national social insurance scheme is usually given from the 17th day of sickness absence (shorter spells are compensated by the employer). To qualify for sickness benefit, occupational disability must be documented by a sick leave certificate issued by a physician (Strømholm et al. 2015). The medical diagnosis on the sick leave certificate is classified according to the International Classification of Primary Care 2.

### Plans for analysis

In line with the proposed research questions, analyses will consider work exposures as predictors for health complaints, sickness absence, and disability retirement. Somatic and mental complaints will also be investigated as potential mediators of the relationship between work exposures and sickness absence/disability. Individual dispositions/attitudes and several work factors (e.g., decision latitude, mastery of work, social support, and leadership) will be included as moderators of associations between work exposures and outcome variables. We will produce both unadjusted and adjusted models. When theoretically applicable, potential confounders for the adjusted models, e.g., demographic variables, will be considered. Analyses that include data on sickness absence will be based on approaches that model count data such as Poisson regression and multi-nominal regression analyses. Analyses of disability retirement will be based on survival data models such as Cox regressions. Complex models on moderator and mediator variables will be analyzed using structural equations models. In addition, logistic regressions, mixed models with repeated measures, and growth-curve analyses will be used. Analyses of reversed associations (strain–stressor relationship) will be conducted when appropriate. A sample size of 14,501 respondents is considered as adequate for determining associations between work factors, sickness absence, and disability.^4^

## Discussion

The current project is set to generate substantial information which will be the subject of multiple scientific articles and reports. The findings will have relevance for, and benefits to, society in that knowledge of which work factors that contribute to health, absence, and participation in/exit from working life, as well as an understanding of mediators and moderators, is central to the development of laws and regulations and to any political decision on measures against absence and early retirement. Furthermore, this knowledge is necessary for decision making and interventions in the organizations and work groups.

The study and its design have several strengths. Through a longitudinal, prospective design, this project performs repeated measurements of work factors, intervening variables, and outcomes. This provides more reliable data on exposures (independent variables) than what has been customary until now. Also, information on variation across time is accounted for. As the survey part builds on the well-established QPS_Nordic (Dallner et al. 2000) as well as on other standardized inventories, the included instruments are psychometrically tested for validity and reliability. Several moderating or mediating factors are measured, allowing the study of social and psychological mechanisms of effects. Registry data are objective data and the data structure allows for multilevel models where individual level data are aggregated to department and organizational levels. Finally, the acquired response rate at the baseline survey assessment is in accordance with the estimated average response rate established in this kind of survey research (Baruch and Holtom 2008).

There are limitations of the planned study. While registry data are objective, the included survey instruments are all self-report measures and the study suffers from the problems that are specific to self-report instruments such as response-set tendencies. Still, the QPS_Nordic_ instrument used in the current study should be fairly insensitive to respondents’ emotions or personality dispositions. QPS_Nordic_-items are constructed with the aim of avoiding emotive content and social desirability bias in that subjects report frequency of occurrence rather than degrees of agreement or satisfaction or stress. Items do not address issues that are inherently negative or positive (Christensen and Knardahl 2012). Although the study sample can be considered as randomized at the individual level as all employees in the included organizations were invited to participate, the sample is not random at an organizational level. Following research which show that non-random and random sample produce quite different results (Nielsen and Einarsen 2008), findings from the present project should be replicated in samples that rely on other kinds of sampling techniques. However, representative data obtained by random sampling in one country may not necessarily be valid for other countries with different compensation systems, industries, or culture. Although the response rate at baseline is at the average for survey research, there is a 50 % non-response which may influence the generalizability of the project. In a previous study, based on the survey data part of this project, which examined attrition from baseline to follow-up it was found that there were few differences in response rate at follow-up between persons with and without health complaints at the baseline measurement (Nielsen and Knardahl 2015b). This suggests that there are few reasons to assume that the sample is obstructed by a healthy worker bias. However, as the response rate increased incrementally with educational level there seems to be a socio-educational bias in response which needs to be considered when interpreting the results from the project.

## Conclusions

In summary, “The new workplace II” study is a major new resource for the investigation of both work- and health related risks for participation and exit from working life. In order to add to the existing knowledge about sickness absence and disability, a main aim of the project is to establish mediating and moderating factors that can explain how and when exposures at the workplace is related to subsequent sickness absence and disability retirement. Consequently, in addition to determining specific risk factors, the findings from the project will generate an understanding of the mechanisms that govern the associations in question. This kind of knowledge is especially important with regard to the development of sound and reliable interventions and measures for reducing sickness absence and increasing the work ability of the population.

## Abbreviations

COR: conservation of resources theory
GSE: generalized self-efficacy
HSCL: Hopkins symptom checklist
IW-agreement: inclusive working life agreement
LOT-R: revised life orientation test
NIOH: The National Institute of Occupational Health
NOU: Norwegian Official Report
QPS_Nordic: General Nordic Questionnaire for Psychological and Social Factors at Work
REC: Regional Committees for Medical and Health Research Ethics
STYRK: Norwegian standard classification of the occupations
WAI: work ability index

## Footnotes

1 http://www.ssb.no/sykefratot_en/.

2 https://www.regjeringen.no/contentassets/7ba70753a7514df58b625f35c27da6fd/no/pdfs/nou201020100013000dddpdfs.pdf.

3 https://www.nav.no/en/Home/About+NAV/Publications/_attachment/263729?_download=true&_ts=14503b8c560.

4 http://www.sample-size.net/sample-size-conf-interval-proportion/.

## References

[CR1] Ala-Mursula L, Vahtera J, Linna A, Pentti J, Kivimaki M (2005). Employee worktime control moderates the effects of job strain and effort-reward imbalance on sickness absence: the 10-town study. J Epidemiol Community Health.

[CR2] Allebeck P, Mastekaasa A (2004). Swedish Council on Technology Assessment in Health Care (SBU), chap 3. Causes of sickness absence: research approaches and explanatory models. Scand J Public Health Suppl.

[CR3] Alvaro C, Lyons RF, Warner G, Hobfoll SE, Martens PJ, Labonte R et al (2010) Conservation of resources theory and research use in health systems. Implement Sci 5:79. doi:10.1186/1748-5908-5-79 10.1186/1748-5908-5-79 PMC2978118 20961445

[CR4] Amanzio M, Corazzini LL, Vase L, Benedetti F (2009). A systematic review of adverse events in placebo groups of anti-migraine clinical trials. Pain.

[CR5] Appelberg K, Romanov K, Heikkilä K, Honkasalo M-L, Koskenvou M (1996). Interpersonal conflict as a predictor of work disability: a follow-up study of 15,348 Finnish employees. J Psychosom Res.

[CR6] Ariens GA, van Mechelen W, Bongers PM, Bouter LM, van der Wal G (2001). Psychosocial risk factors for neck pain: a systematic review. Am J Ind Med.

[CR7] Baruch Y, Holtom BC (2008). Survey response rate levels and trends in organizational research. Hum Relat.

[CR8] Blekesaune M, Solem PE (2005). Working conditions and early retirement—a prospective study of retirement behavior. Res Aging.

[CR9] Carver CS (1997). You want to measure coping but your protocol’s too long: consider the brief COPE. Int J Behav Med.

[CR10] Carver CS, Scheier MF, Weintraub JK (1989). Assessing coping strategies: a theoretically based approach. J Pers Soc Psychol.

[CR11] Christensen JO, Knardahl S (2010). Work and neck pain: a prospective study of psychological, social, and mechanical risk factors. Pain.

[CR12] Christensen JO, Knardahl S (2012). Work and back pain: a prospective study of psychological, social and mechanical predictors of back pain severity. Eur J Pain.

[CR13] Christensen JO, Knardahl S (2012). Work and headache: a prospective study of psychological, social, and mechanical predictors of headache severity. Pain.

[CR14] Conchie SM (2013). Transformational leadership, intrinsic motivation, and trust: a moderated-mediated model of workplace safety. J Occup Health Psychol.

[CR15] Dallner M, Elo A-L, Gamberale F, Hottinen V, Knardahl S, Lindström K (2000). Validation of the General Nordic Questionnaire (QPSNordic) for psychological and social factors at work.

[CR16] Derogatis LR, Lipman RS, Rickels K, Uhlenhuth EH, Covi L (1974). The Hopkins symptom checklist (HSCL): a self report symptom inventory. Behav Sci.

[CR17] Dragano N, Schneider L (2011). Work related psychosocial factors and the risk of early disability pensioning: a contribution to assessing the need for rehabilitation. Rehabilitation.

[CR18] Emberland JS, Knardahl S (2015). Contribution of psychological, social, and mechanical work exposures to low work ability: a prospective study. J Occup Environ Med.

[CR19] Finne LB, Christensen JO, Knardahl S (2014). Psychological and social work factors as predictors of mental distress: a prospective study. PLoS One.

[CR20] Folkman S, Moskowitz JT (2004). Coping: pitfalls and promise. Annu Rev Psychol.

[CR21] Goldberg LR, Mervielde I, Deary I, De Fruyt F, Ostendorf F (1999). A broad-bandwidth, public domain, personality inventory measuring the lower-level facets of several five-factors model. Personality psychology in Europe.

[CR22] Hobfoll SE (1989). Conservation of resources: a new attempt at conceptualizing stress. Am Psychol.

[CR23] Ilmarinen J, Tuomi K, Klockars M (1997). Changes in the work ability of active employees over an 11-year period. Scand J Work Environ Health.

[CR24] Indregard AM, Ihlebaek CM, Eriksen HR (2013). Modern health worries, subjective health complaints, health care utilization, and sick leave in the Norwegian working population. Int J Behav Med.

[CR25] Johannessen H, Tynes T, Sterud T (2013) Effects of occupational role conflict and emotional demands on subsequent psychological distress. A 3-year follow-up study of the general working population in Norway. J Occup Environ Med 55(6):605–613. doi:10.1097/JOM.0b013e3182917899 10.1097/JOM.0b013e3182917899 23722939

[CR26] Johns G (2010). Presenteeism in the workplace: a review and research agenda. J Organ Behav.

[CR27] Kivimaki M, Forma P, Wikstrom J, Halmeenmaki T, Pentti J, Elovainio M (2004). Sickness absence as a risk marker of future disability pension: the 10-town study. J Epidemiol Community Health.

[CR28] Kjeldsberg M, Tschudi-Madsen H, Dalen I, Straand J, Bruusgaard D, Natvig B (2013). Symptom reporting in a general population in Norway: results from the Ullensaker study. Scand J Prim Health Care.

[CR29] Knardahl S, Johannessen H, Sterud T, Härmä M, Rugulies R, Seitsamo J et al A systematic review of the contribution from psychological, social, and organizational factors at work to risk of disability retirement. Plos One **(submitted)** 10.1186/s12889-017-4059-4 PMC5299735 28178966

[CR30] Krane L, Johnsen R, Fleten N, Nielsen CV, Stapelfeldt CM, Jensen C (2014). Sickness absence patterns and trends in the health care sector: 5-year monitoring of female municipal employees in the health and care sectors in Norway and Denmark. Hum Resour Health.

[CR31] Kristensen TS (1991). Sickness absence and work strain among Danish slaughterhouse workers—an analysis of absence from work regarded as coping behavior. Soc Sci Med.

[CR32] Kristensen TS, Borritz M, Villadsen E, Christensen KB (2005). The Copenhagen burnout inventory: a new tool for the assessment of burnout. Work Stress.

[CR33] Kuper H, Marmot M (2003). Job strain, job demands, decision latitude, and risk of coronary heart disease within the Whitehall II study. J Epidemiol Community Health.

[CR34] Lazarus RS, Folkman S (1984). Stress, appraisal and coping.

[CR35] Leeuw M, Goossens MEJB, Linton SJ, Crombez G, Boersma K, Vlaeyen JWS (2007). The fear-avoidance model of musculoskeletal pain: current state of scientific evidence. J Behav Med.

[CR36] Lund T, Labriola M, Christensen KB, Bultmann U, Villadsen E (2006). Physical work environment risk factors for long term sickness absence: prospective findings among a cohort of 5357 employees in Denmark. BMJ.

[CR37] Marmot M, Feeney A, Shipley M, North F, Syme SL (1995). Sickness absence as a measure of health-status and functioning—from the UK Whitehall-II Study. J Epidemiol Community Health.

[CR38] Michie S, Williams S (2003). Reducing work related psychological ill health and sickness absence: a systematic literature review. Occup Environ Med.

[CR49] MoHaC Services (2014) Internasjonal sammenligning av sykefraværet (International comparison of sickness absence rates). Proba samfunnsanalyse 2014. Report No.: 2014-05, Oslo, Norway

[CR39] Nguyen H, Groth M, Johnson A (2013). When the going gets tough, the tough keep working: impact of emotional labor on absenteeism. J Manag.

[CR40] Niedhammer I, Goldberg M, Leclerc A, David S, Bugel I, Landre MF (1998). Psychosocial work environment and cardiovascular risk factors in an occupational cohort in France. J Epidemiol Community Health.

[CR41] Niedhammer I, Chastang JF, Sultan-Taieb H, Vermeylen G, Parent-Thirion A (2013). Psychosocial work factors and sickness absence in 31 countries in Europe. Eur J Public Health.

[CR42] Nielsen MB, Einarsen S (2008). Sampling in research on interpersonal aggression. Aggress Behav.

[CR43] Nielsen MB, Knardahl S (2014). Coping strategies: a prospective study of patterns, stability, and relationships with psychological distress. Scand J Psychol.

[CR44] Nielsen MB, Knardahl S (2015). Is workplace bullying related to the personality traits of victims? A two year prospective study. Work Stress.

[CR45] Nielsen MB, Knardahl S (2015). The healthy worker effect: do health problems predict participation rates in, and the results of, a follow-up survey?. Int Arch Occup Environ Health.

[CR46] Nielsen MB, Tvedt SD, Matthiesen SB (2012). Prevalence and occupational predictors of psychological distress in the offshore petroleum industry: a prospective study. Int Arch Occup Environ Health.

[CR47] Scheier MF, Carver CS, Bridges MW (1994). Distinguishing optimism from neuroticism (and trait anxiety, self-mastery, and self-esteem)—a reevaluation of the life orientation test. J Pers Soc Psychol.

[CR48] Schwarzer R, Jerusalem M, Weinman J, Wright S, Johnston M (1995). Generalized self-efficacy scale. Measures in health psychology: a user’s portfolio. Causal and control beliefs.

[CR50] Steers RM, Rhodes SR (1978). Major influences on employee attendance: a process model. J Appl Psychol.

[CR51] Steingrimsdottir OA, Vollestad NK, Roe C, Knardahl S (2004). Variation in reporting of pain and other subjective health complaints in a working population and limitations of single sample measurements. Pain.

[CR52] Sterud T (2014). Work-related gender differences in physician-certified sick leave: a prospective study of the general working population in Norway. Scand J Work Environ Health.

[CR53] Strømholm T, Pape K, Ose SO, Krokstad S, Bjorngaard JH (2015). Psychosocial working conditions and sickness absence in a general population: a cohort study of 21,834 workers in Norway (The HUNT Study). J Occup Environ Med.

[CR54] Tynes T, Johannessen HA, Sterud T (2013). Work-related psychosocial and organizational risk factors for headache: a 3-year follow-up study of the general working population in Norway. J Occup Environ Med.

[CR55] Vleeshouwers J, Knardahl S, Christensen JO (2015) Effects of psychological and social work factors on self-reported sleep disturbance and difficulties initiating sleep. Sleep 10.5665/sleep.5638 PMC4791617 26446114

[CR56] Wannstrom I, Peterson U, Asberg M, Nygren A, Gustavsson JP (2009). Psychometric properties of scales in the General Nordic Questionnaire for Psychological and Social Factors at Work (QPSNordic): confirmatory factor analysis and prediction of certified long-term sickness absence. Scand J Psychol.

[CR57] Zapf D, Vogt C, Seifert C, Mertini H, Isic A (1999). Emotion work as a source of stress: the concept and development of an instrument. Eur J Work Org Psychol.

